# Latent profiles of occupational stress and their association with premenstrual syndrome: a cross-sectional study of Chinese nurses

**DOI:** 10.3389/fpubh.2026.1683290

**Published:** 2026-01-27

**Authors:** Yuecong Wang, Xin Wang, Chengcai Wen, Xiwen Yang, Zhikun Zhao, Jiawen Zhou, Wenya Wang, Hua Tao, Lili Chen

**Affiliations:** 1 Department of Neurosurgery, The Second People’s Hospital of Huai’an, The Affiliated Huai’an Hospital of Xuzhou Medical University, Huai'an, Jiangsu, China; 2 Department of Emergency, Huaian Hospital of Huaian City, Huaian, Jiangsu, China; 3 Department of Nursing, Jinzhou Medical University, Jinzhou, China; 4 Department of Urology, The Second People’s Hospital of Huai’an, The Affiliated Huai’an Hospital of Xuzhou Medical University, Huai'an, Jiangsu, China; 5 Department of ICU, The Second People’s Hospital of Huai’an, The Affiliated Huai’an Hospital of Xuzhou Medical University, Huai'an, Jiangsu, China; 6 Department of Thoracic Surgery, The Second People’s Hospital of Huai’an, The Affiliated Huai’an Hospital of Xuzhou Medical University, Huai'an, Jiangsu, China; 7 Department of Oncology, The Second People’s Hospital of Huai’an, The Affiliated Huai’an Hospital of Xuzhou Medical University, Huai'an, Jiangsu, China; 8 Department of Nursing, The Second People’s Hospital of Huai’an, The Affiliated Huai’an Hospital of Xuzhou Medical University, Huai'an, Jiangsu, China

**Keywords:** latent profile analysis, nurse, occupational stress, premenstrual syndrome, type

## Abstract

**Objectives:**

Occupational stress in nursing is a critical issue that can have significant implications for both workforce stability and personal health. This study aimed to identify subgroups of occupational stress among Chinese female clinical nurses using latent profile analysis, compare sociodemographic differences across these subgroups, and examine their associations with premenstrual syndrome (PMS).

**Methods:**

A cross-sectional study was conducted among female nurses in tertiary hospitals in Huai’an City, Jiangsu Province, China, from November to December 2023. We recruited participants via convenience sampling, and 400 valid questionnaires were collected. Data were collected using a researcher-developed general information questionnaire, the standardized Chinese Nurses Stressor Scale (35 items), and the Premenstrual Syndrome Scale. Latent profile analysis (LPA) was performed with Mplus 8.0 to identify occupational stress subtypes. Sociodemographic predictors of these subtypes were explored using chi-square tests and multivariate logistic regression in SPSS 25.0. The association between stress subtypes and PMS symptoms was assessed using ANOVA. A *p*-value of < 0.05 was considered statistically significant for all analyses.

**Results:**

Three clinical female nurse occupational stress subtypes were identified: overall low-stress (38.3%, *n* = 153), moderate stress–slight overload care (38.5%, *n* = 154), and high stress–overload nursing and career development challenge (23.2%, *n* = 93). Age, years of work experience, and monthly night shifts were key influencing factors. The results revealed a statistically significant difference in PMS scores across the stress groups (*p* < 0.001). Specifically, nurses in the high stress–overload nursing and career development challenge group faced a significantly higher risk for PMS, whereas those in the overall low-stress group exhibited the fewest related symptoms.

**Conclusion:**

This study identified significant heterogeneity in occupational stress among clinical female nurses, categorized into three distinct subtypes differing in stress levels and demographic characteristics. These findings highlight the importance of considering individual differences when developing interventions to address occupational stress. The study advocates for the implementation of intervention strategies targeting different types of stress in nursing education and organizational reform to better support nurses in fulfilling their responsibilities.

## 1 Introduction

Occupational stress is a state of physical and mental stress in a practitioner’s work environment resulting from an imbalance between occupational demands and personal abilities or perceptions (1). In the nursing profession, occupational stress in nurses is specifically characterized by the multiple burdens they bear in their daily work, leading to subjective discomfort (2, 3). Relevant study has shown (4) that, globally, more than 50% of the nurse population is in a state of chronic high stress in their professional environment. Further research revealed (5–7) that nurses experience stress levels that are 29% higher than those of the general population and that the situation is particularly dire for female nurses. Not only do they have to cope with traditional occupational stressors, they also often suffer from gender bias and face greater challenges in balancing work and family responsibilities. These additional stressors further aggravate their physical and mental burdens. Moderate work stress can stimulate nurses’ creativity and increase their productivity. However, when stress exceeds the coping range, it can lead to negative attitudes and behaviors that affect professional performance. For female nurses, this effect is more pronounced because of the overlap of social expectations, gender roles, and family responsibilities. Prolonged unrelieved stress can lead to burnout in the form of emotional exhaustion, depersonalization, and reduced achievement (8) and may also trigger anxiety and depression, affecting mental health and job satisfaction (6, 9, 10). Female nurses may face greater difficulties in coping with these stresses because of inadequate social support systems, gender discrimination, and unfair treatment in the workplace. Nurses who are chronically exposed to high levels of occupational stress are more likely to develop a tendency to leave their jobs, affecting their personal career development and the quality of services provided by healthcare organizations. This phenomenon is particularly prominent among female nurses, leading to attrition and a decline in the quality of the nursing workforce. Therefore, identifying and managing occupational stress among nurses, especially female nurses, is critical to maintaining the stability of the nursing team and improving the quality of healthcare services.

Chegini (7) explored the occupational stress of nurses in intensive care units in depth, and their findings revealed that up to 64% of nurses showed a strong tendency to leave their jobs due to insufficient clinical experience, heavy responsibilities, and complex interpersonal relationships, which profoundly revealed a strong link between occupational stress and the intention to leave. Furthermore, Alruwaili (11) explored the current work situation of nurses in the emergency department in detail and reported that long working hours, complex work environments, heavy workloads, inadequate staffing, and poor salary packages collectively constituted the major sources of occupational stress among nurses in the emergency department. In addition, a study by Chaudhari (12) revealed that occupational stress among clinical nurses peaks at approximately 10 years of employment, whereas nurses with relatively little experience, whose operational proficiency has not yet been fully developed, are faced with even more prominent occupational stress when dealing with emergencies. Together, these studies reveal the major sources of occupational stress faced by nurses in different clinical scenarios, including multidimensional factors such as the work environment, experience accumulation, interpersonal interactions, and workload.

Critically, the physiological and psychological burden of occupational stress may not exist in isolation but can interact with and exacerbate other health conditions, particularly those sensitive to stress. There is a growing body of evidence suggesting a bidirectional relationship between chronic stress and gynecological health (1, 9, 13). Specifically, the experience of chronic occupational stress can dysregulate the hypothalamic–pituitary–adrenal (HPA) axis and hormonal balance, which are key pathways implicated in the severity of premenstrual symptoms (14, 15). Conversely, the recurring physical discomfort and emotional lability associated with premenstrual syndrome (PMS) can impair an individual’s coping resources, potentially lowering their threshold for perceiving and being affected by workplace stressors (1). This relationship is supported by specific pathways. Physiologically, chronic stress can disrupt cortisol rhythms and sensitize neuroendocrine circuits, amplifying reactivity to hormonal fluctuations. Psychosocially, PMS symptoms like pain and irritability deplete coping resources, lowering tolerance for workplace demands. This creates a vicious cycle where stress worsens PMS symptoms, which in turn amplifies the perception of stress, ultimately compromising well-being and professional performance (1). This interplay is particularly salient for female nurses, who navigate a high-stress profession while also managing cyclical physiological challenges.

PMS is one of the gynecological disorders in China, which characterized by cyclical physical and psychological symptoms, such as headaches, sleep disturbances, social withdrawal, and mood swings, which typically occur during the luteal phase of a woman’s menstrual cycle, i.e., between the first 7 and 14 days of menstruation, and then gradually diminish (16). A review of the literature revealed (17, 18) that PMS affects between 25 and 70% of women globally and that this symptom cluster is diverse and has a negative impact on women’s work performance. Most women of childbearing age experience one or more emotional or physical symptoms during the premenstrual phase of the menstrual cycle. Among these patients, between 3 and 10% experience moderate to severe symptoms, which are often accompanied by significant distress or dysfunction (19). In addition, previous studies on the prevalence of PMS and its associated factors have focused on adult student populations (17, 19, 20); however, there is a relative paucity of research on specific occupational groups, including nurses.

Occupational stress among female nurses is a multidimensional and complex issue. Most prior research on the stress-PMS relationship has relied on variable-centered, linear models that examine average associations between stress levels and outcomes (1, 16, 21). Although this approach holds reference value, it assumes population homogeneity and overlooks the potential existence of heterogeneous subgroups with unique stressor characteristics. Consequently, critical gaps remain in understanding the association between specific occupational stress patterns and their differential characteristics within nursing populations and PMS. To address this gap, our study adopts a novel, person-centered approach using latent profile analysis (LPA). Unlike variable-centered approaches, LPA identifies homogeneous latent subgroups within heterogeneous populations based on individuals’ response patterns across multiple stress dimensions (22). This allows us to move beyond asking “how much” stress is linked to PMS, to instead investigate “what patterns” of stress carry the highest risk, thereby directly targeting the heterogeneity ignored in prior linear models. Furthermore, there is a distinct lack of person-centered research exploring occupational stress among female nurses. The categorization of subtypes of occupational stress among female nurses and the associations between different subtypes and PMS have not been extensively and thoroughly researched. Therefore, this study aims to: (i) identify different profiles of occupational stress and their predictors among Chinese female nurses and (ii) analyze the complex relationships between different stress profiles and PMS. Through these studies, we expect to provide a more accurate scientific basis for understanding and coping with occupational stress among female nurses.

## 2 Methods

### 2.1 Study design

This study applied a cross-sectional design and was conducted and reported in strict accordance with the STROBE guidelines.

### 2.2 Study setting and sample size

In this study, the convenience sampling method was used to select nurses in tertiary hospitals in Huai’an city, Jiangsu Province, as the study population. The study survey was conducted from November to December 2023. The inclusion criteria were as follows: (i) female nurses who were qualified as practicing nurses; (ii) aged 18–45 years; (iii) had regular menstrual cycles; and (iv) provided voluntary and informed consent to participate in the study. The exclusion criteria were as follows: (i) were menopausal; (ii) were pregnant; and (iii) had used hormonal, contraceptive, or other medications in the last 3 months. The total number of variables involved in this study was 56. According to the methodology recommended by Kendall (23), the initial estimate of the sample size should be at least 5–10 times the number of variables. Considering the possibility of convenience sampling error and the expected 10% sample attrition, the sample size required for this study ranged from 312 to 623. On this basis, 450 questionnaires were distributed during the study period. A total of 50 questionnaires were excluded, resulting in 400 valid questionnaires for analysis (effective response rate: 88.9%). The excluded questionnaires comprised: (i) 35 incomplete questionnaires with substantial missing data, and (ii) 15 invalid questionnaires were identified through rigorous screening for unqualified responses, such as straight-line or patterned answers. This process and the resulting valid questionnaire recovery rate of 88.9% help to ensure the representativeness of the data and the reliability of the study.

### 2.3 Measures

#### 2.3.1 General information questionnaire

It was designed by the researcher and included age, marital status, education, professional title, years of work experience, clinical unit, number of night shifts per month, career establishment, and average monthly income.

#### 2.3.2 Chinese nurses’ stressor scale (CNSS)

The scale was originally developed by Li et al. (24) and has since become the most commonly used tool for assessing occupational stress among Chinese nurses. In its development and validation study, the scale demonstrated excellent psychometric properties, with a reported Cronbach’s alpha of 0.980 and satisfactory construct validity, confirming its robustness for measuring occupational stress. The scale is rich in content, with a total of 35 items, which are carefully divided into 5 dimensions: nursing profession and work, time allocation and workload, work environment and resources, patient care, and management and interpersonal relationships. The assessment was conducted on a 4-point Likert scale ranging from 1 to 4, corresponding to ‘strongly disagree’ to ‘strongly agree’, with a total score ranging from 35 to 140, with higher scores representing more significant stress levels. In this study, the Cronbach’s alpha of the scale was 0.932, further supporting its reliability and validity in our sample.

#### 2.3.3 Premenstrual syndrome scale (PMSS)

The scale was developed by Zhao (25) on the basis of Bancroft’s diagnostic criteria (26) and is intended to serve as a simple and quick screening tool for PMS. In the present study, the Cronbach’s alpha coefficient was 0.856, indicating good internal consistency. The scale uses 12 physical and psychological symptoms associated with PMS to assess symptoms experienced by women in the 14 days before their most recent menstrual period. The scale is based on a 4-point Likert-type scale ranging from 0 to 3, with a total score ranging from 0 to 36. A higher total score on this scale indicates more severe premenstrual symptoms.

### 2.4 Data collection

Before the official launch of the study, the relevant study plan was approved by the hospital management and the person in charge of the study to ensure that the content of the study followed strict ethical principles. The questionnaires were distributed in a face-to-face paper version to ensure that the participants had direct access to the questionnaires and could begin completing them immediately. At the start of the questionnaire distribution, the investigator provided clear instructions to the participants and explained in detail the main content of the questionnaire to ensure that the clinical nurses fully understood the questionnaire to conduct an accurate and objective self-assessment. To ensure data quality, we established minimum completion times based on pre-test results. Surveys completed within unreasonably short periods are flagged for re-examination. Additionally, we implemented procedural checks during field collection, including: (i) verifying the completeness of all survey responses; (ii) examining surveys for conspicuous response patterns; and (iii) confirming logical consistency within relevant items. Upon completion of the questionnaire, the investigator immediately retrieved the questionnaire and checked it on the spot to ensure the timeliness and completeness of the data, thus safeguarding the validity and accuracy of the study data.

### 2.5 Data analysis

In this study, SPSS 25.0 software was used for data analysis, and the count information was described by frequency (%). MPLUS 8.0 software was used to construct the LPA. On the basis of the general research steps (22), the specific implementation process of exploratory LPA was developed in this study: (i) Model construction: starting from the baseline model, the number of profiles was gradually increased to establish the potential profile model. (ii) Model comparison and selection: Based on the parameter estimation and fitting indices of each model, the comparison and selection among models are carried out. (iii) Profile naming: Based on the mean response of the entries, the profiles are categorized and named according to the performance of each profile on different indicators. (iv) Classification attribution: Classification is performed to determine the attribution category of each observation.

Single-factor analysis was used to screen statistically significant independent variables, and multivariate logistic regression analyses were performed to explore the predictors of occupational stress among female nurses with different LPA profiles. In addition, the differences between different profiles and PMS were explored via ANOVA, with *p* < 0.05 indicating a statistically significant difference.

The fit test indices of the LPA model include the Akaike information criterion (AIC), Bayesian information criterion (BIC), and adjusted Bayesian information criterion (aBIC), and the smaller the values of these indices are, the better the fit of the model (27). Moreover, the differences in fit between potential categories were compared via the Lo–Mendell–Rubin (LMR) and bootstrapped likelihood ratio test (BLRT), which indicated that the model with K categories was better than the model with K-1 categories when *p* < 0.05. The value of entropy ranges from 0 to 1; the closer the value is to 1, the higher the classification accuracy, and models with entropy values over 0.8 are considered acceptable (28, 29). In the selected model, if the mean posterior probability for each category exceeds 0.85, it indicates that the classification of the profile exhibits high certainty. The final naming of each profile is based on data-driven logic and determined through analysis and interpretation of the unique response pattern characteristics of each subgroup.

### 2.6 Ethics approval and consent to participate

This study was conducted in accordance with the principles of the Declaration of Helsinki and received formal ethical approval from the Ethics Committee of Jinzhou Medical University (Approval Number: JZMULL2023105). Prior to their participation in the study, written informed consent was obtained from all individual participants involved.

## 3 Results

### 3.1 Demographic characteristics of the participants

As detailed in the Methods section, 450 participants were recruited. The final analytical sample consisted of 400 nurses. This was after the exclusion of 50 questionnaires, which comprised 35 cases with substantial missing data and 15 cases with invalid response patterns, resulting in an effective response rate of 88.9%. Among these participants, 50.7% were over 30 years of age, and 66.3% were married. Among them, 72.3% of the female nurses had a bachelor’s degree or higher, 47.3% of the participants held the title of nurse practitioner, and 67.3% of the nurses had ≤10 years of work experience. Among the nursing specialties, 40.5% were engaged in internal medicine nursing, and 86.0% of these nurses were not included in the career establishment. In addition, the proportion of nurses earning 5,000 RMB or more per month was 67.5, and 60.5% of female nurses were required to work five or more night shifts per month. More specific information about the participants is detailed in Table 1.

**Table 1:** Demographic characteristics of the participants.

Variable	*N*	*%*
Age		
18–30	197	49.3
>30	203	50.7
Marital status		
Unmarried	116	29.0
Married	265	66.3
Divorced or widowed	19	4.8
Educational		
Junior college degree and below	111	27.8
Bachelor’s degree and above	289	72.3
Professional title		
Nurse	53	13.3
Nurse Practitioner	189	47.3
Supervisor Nurse	129	32.3
Associate Nurse Practitioner and above	29	7.2
Years of work experience (Years)		
≤10	269	67.3
>10	131	32.8
Clinical unit		
Medicine	162	40.5
Surgical	157	39.3
Emergency	28	7.0
Gynecology and pediatrics	30	7.5
ICU	23	5.8
Number of night shifts per month		
≤5	158	39.5
>5	242	60.5
With a career establishment		
Yes	56	14.0
No	344	86.0
Average monthly income (RMB)		
<5,000	130	32.5
≥5,000	270	67.5

M: mean; SD: standard deviation.

### 3.2 Latent profile analysis and naming of occupational stress among female nurses

The results of the data analysis are shown in Table 2. As the number of model profiles increases, the values of the AIC, BIC, and aBIC gradually decrease. When the number of model profiles reaches 3, the decreasing trend of these indicators starts to slow, which indicates that the fitting advantage of the model gradually weakens. Meanwhile, the entropy values of all the profiles are greater than 0.8, which indicates that the classification model has a good classification effect, especially for profile 3, and the entropy value reaches the highest point of 0.948, which further confirms the superiority of the model in this profile. In addition, the LMR test results were significant for 2 and 3 profiles and no longer significant for 4 or more profiles. Together, these findings support the rationale for choosing the 3-profile as the optimal model.

**Table 2:** Fitting indices for potential profiles of occupational stress among female nurses.

Model	Loglikelihood	AIC	BIC	aBIC	Entropy	LMR	BLRT	Profile probability
1-profile	−18983.449	38106.897	38386.300	38164.185	N/A	N/A	N/A	1
2-profile	−17284.560	34781.119	35204.214	34867.869	0.945	<0.001	<0.001	0.49/0.51
**3-profile**	**−16782.561**	**33849.121**	**34415.909**	**33965.334**	**0.948**	**<0.001**	**<0.001**	**0.38/0.39/0.23**
4-profile	−16649.651	33655.302	34365.782	33800.976	0.921	0.730	<0.001	0.30/0.32/0.25/0.13
5-profile	−16559.402	33546.805	34400.978	33721.942	0.909	0.252	<0.001	0.27/0.16/0.28/0.20/0.09

The model solution chosen is bolded.

**Figure 1:**
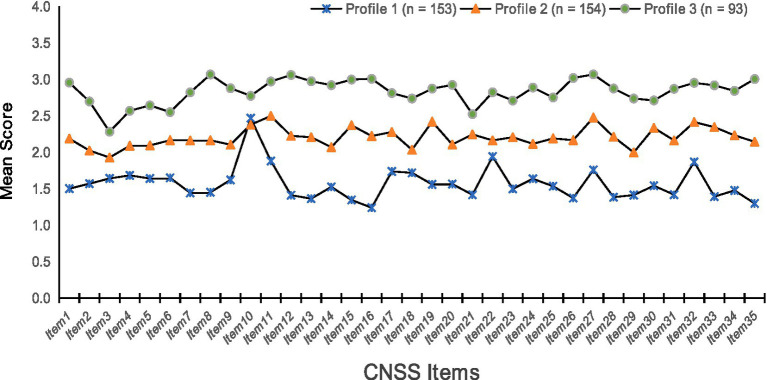
Three potential profiles of occupational stress in female clinical nurses. F1: Nursing profession and work (Items 1–7); F2: Time allocation and workload (Items 8–12); F3: Work environment and resources (Items 13–15); F4: Patient care (Items 16–26); F5: Management and interpersonal relationships (Items 27–35).

**Figure 2:**
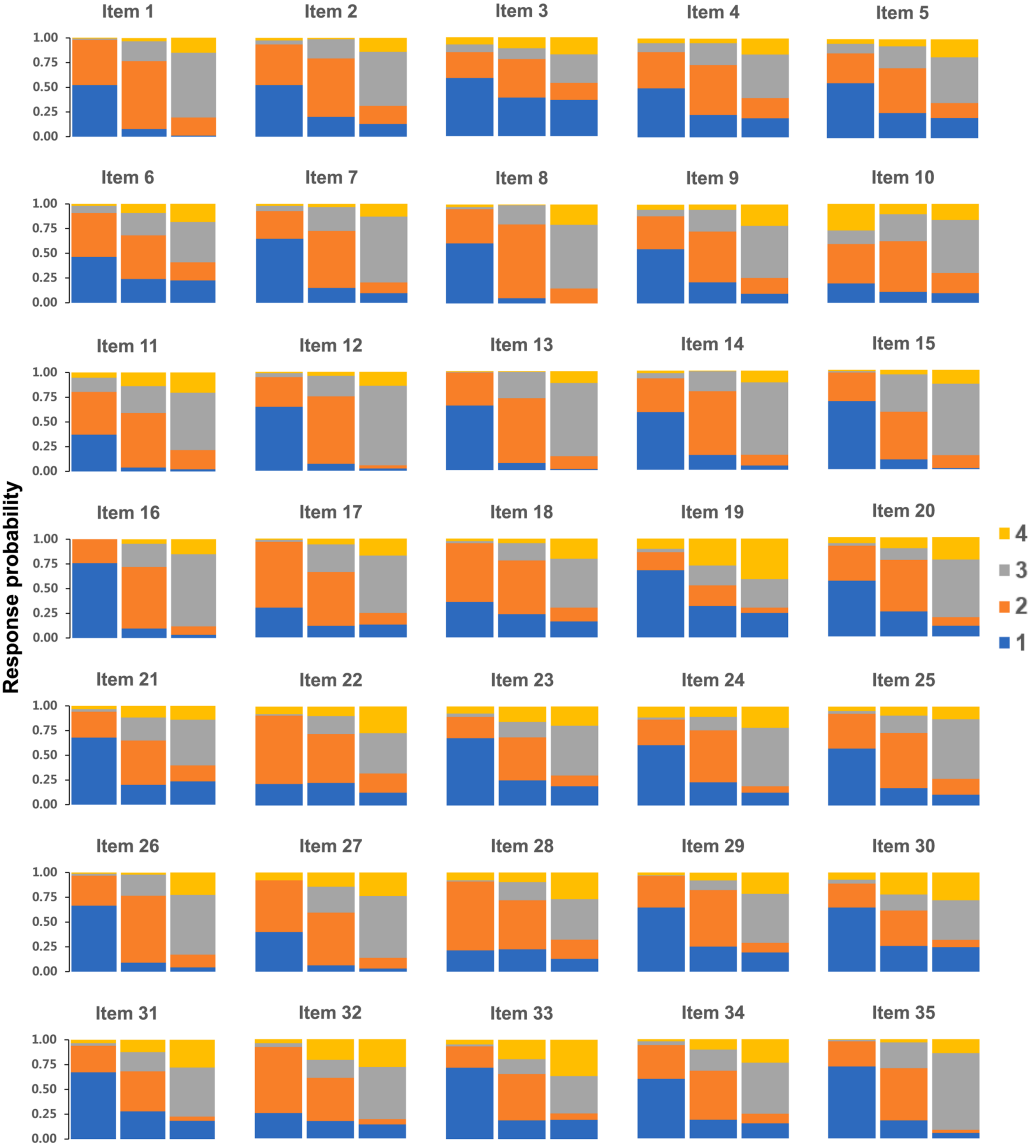
Response probabilities of the 35 items of occupational stress among female nurses in the 3-profile model.

On the basis of the results of the latent profile analysis, the scores of the three profiles and response probabilities for the 35 items of the Occupational Stress Scale were plotted, as shown in Figures 1 and 2. The three profiles were rationally named by analyzing the mean values of the scores for each item and the fluctuations in the line graphs.

Profile 1 (Overall Low-Stress Group): Comprised 38.3% (*n* = 153) of the sample, characterized by low mean scores across most stress dimensions.

Profile 2 (Moderate Stress-Slight Overload Care Group): Comprised 38.5% (*n* = 154), characterized by moderate mean scores overall, with slightly elevated scores on care-related items.

Profile 3 (High Stress-Overload Nursing and Career Development Challenge Group): Comprised 23.2% (*n* = 93), characterized by high mean scores overall, with particularly elevated scores in ‘patient care and responsibility’ and ‘career development and identity’ dimensions.

### 3.3 Single-factor analysis of latent profile analysis of occupational stress among female nurses

The results revealed statistically significant (*p* < 0.05) differences between the three profiles in terms of age, years of work experience, and number of night shifts per month. In contrast, the differences in occupational stress among female clinical nurses in the three profiles of marital status, education level, professional title, clinical department, average monthly income, and career establishment were not statistically significant (*p* > 0.05). Details of the results are shown in Table 3.

**Table 3:** Single-factor analysis of different latent profile analyses of occupational stress among female nurses.

Variable	Overall low- stress group (*n* = 153)	Moderate stress -slight overload care group (*n* = 154)	High stress-overload nursing and career development challenge group (*n* = 93)	*X^2^*	*p*
Age				59.651	<0.001
18–30	38 (19.3)	102 (51.8)	57 (28.9)		
>30	115 (56.7)	52 (25.6)	36 (17.7)		
Marital status				2.557	0.634
Unmarried	41 (35.3)	49 (42.2)	26 (22.4)		
Married	107 (40.4)	96 (36.2)	62 (23.4)		
Divorced or widowed	5 (26.3)	9 (47.4)	5 (26.3)		
Educational				1.090	0.580
Junior college degree and below	47 (42.3)	40 (36.0)	24 (21.6)		
Bachelor’s degree and above	106 (36.7)	114 (39.4)	69 (23.9)		
Professional title				8.546	0.201
Nurse	18 (34.0)	22 (41.5)	13 (24.5)		
Nurse Practitioner	62 (32.8)	80 (42.3)	47 (24.9)		
Supervisor Nurse	57 (44.2)	45 (34.9)	27 (20.9)		
Associate Nurse Practitioner and above	16 (55.2)	7 (24.1)	6 (20.7)		
Years of work experience				73.489	<0.001
	64 (23.8)	131 (48.7)	74 (27.5)		
	89 (67.9)	23 (17.6)	19 (14.5)		
Clinical unit				10.266	0.247
Medicine	53 (32.7)	61 (37.7)	48 (29.6)		
Surgical	61 (38.9)	65 (41.4)	31 (19.7)		
Emergency	15 (53.6)	8 (28.6)	5 (17.9)		
Gynecology and pediatrics	15 (50.0)	10 (33.3)	5 (16.7)		
ICU	9 (39.1)	10 (43.5)	4 (17.4)		
Number of night shifts per month				22.914	<0.001
≤5	83 (52.5)	49 (31.0)	26 (16.5)		
>5	70 (28.9)	105 (43.4)	67 (27.7)		
With a career establishment				4.425	0.109
Yes	20 (35.7)	17 (30.4)	19 (33.9)		
No	133 (38.7)	137 (39.8)	74 (21.5)		
Average monthly income (RMB)				10.346	0.006
<5,000	40 (30.8)	60 (46.2)	30 (23.1)		
≥5,000	113 (41.9)	94 (34.8)	63 (23.3)		

### 3.4 Multivariate logistic regression analysis of latent profile analysis of occupational stress in female nurses

In this study, the latent profile of occupational stress among female nurses was used as the dependent variable, and statistically significant variables in the single-factor analysis (*p* < 0.05) were selected as the independent variables in the multivariate logistic regression analysis. The results of the analysis revealed that in the comparison of the moderate stress–slight overload care group with the overall low-stress group, nurses aged 18–30 years (OR = 2.328, 95% CI: 1.223--4.432) and with ≤ 10 years of work experience (OR = 3.994, 95% CI: 1.988--8.021) were more likely to belong to the moderate stress–slight overload care group, whereas nurses with ≤ 5 night shifts per month (OR = 0.596, 95% CI: 0.356–0.997) were more likely to belong to the overall-low stress group. In a comparison of the high stress-overload nursing and career development challenge group with the overall low-stress group, nurses aged 18--30 years (OR = 2.162, 95% CI: 1.037--4.506) and with ≤10 years of work experience (OR = 2.738, 95% CI: 1.247--6.015) were more likely to belong to the high stress-overload nursing and career development challenge group, whereas nurses with ≤ 5 night shifts monthly (OR = 0.463, 95% CI: 0.257--0.832) were more likely to belong to the overall low-stress group. The results of the specific regression analyses are detailed in Table 4.

**Table 4:** Multivariate logistic regression analysis of latent profile analysis of occupational stress among female nurses.

Variable	Overall low-Stress group (Ref)					
	Moderate Stress -Slight Overload Care Group			High Stress-Overload Nursing and Career Development Challenge Group		
	OR	95% CI	*p* value	OR	95% CI	*p* value
Age						
18–30	2.328	1.223–4.432	0.010	2.162	1.037–4.506	0.040
Ref: >30						
Years of working experience						
≤10	3.994	1.988–8.021	<0.001	2.738	1.247–6.015	0.012
Ref: >10						
Number of night shifts per month						
≤5	0.596	0.356–0.997	0.048	0.463	0.257–0.832	0.010
Ref: >5						

### 3.5 Relationships between profiles of occupational stress and PMS among female nurses

The results revealed that the difference in the total PMS scores of female nurses in different occupational stress groups was statistically significant (*F* = 50.564, *p* < 0.001). Specifically, female nurses in the high stress-overload nursing and career development group (10.20 ± 5.95) had the highest total PMS score. In contrast, female nurses in the overall low-stress group (4.62 ± 3.94) had the lowest total PMS score. See Table 5 for details.

**Table 5:** Relationships between profiles of occupational stress and PMS among female nurses.

Variable	Total score for premenstrual syndrome
Overall low-Stress group	4.62 ± 3.94
Moderate Stress-Slight Overload Care Group	7.43 ± 3.26
High Stress-Overload Nursing and Career Development Challenge Group	10.20 ± 5.95
*F*	50.564
*p*	<0.001
*PostHoc*	3>2>1

## 4 Discussion

The primary aims of this study were to identify distinct latent profiles of occupational stress and their influencing factors among Chinese female clinical nurses, and to explore their association with PMS. The latent profile analysis identified three distinct occupational stress profiles among Chinese female clinical nurses: an overall low-stress group (38.3%), a moderate stress-slight overload care group (38.5%), and a high stress-overload nursing and career development challenge group (23.2%). The excellent classification accuracy (entropy = 0.948) and statistical indicators support the validity of this three-profile structure. We posit that these profiles represent meaningful subgroups with different stress experiences and intervention needs, moving beyond the traditional variable-centered approach to capture the holistic pattern of stress experiences (22, 28). This categorization is structurally similar to the findings of Luo et al. (9) but differs in specifics and nomenclature. Luo et al.’s study focused on occupational stress and metabolic dysfunction associated with steatohepatitis, whereas the present study focused on the relationship between occupational stress and PMS. This difference may be due to differences in the research background, sample characteristics, and assessment tools used (30). The present study not only systematically revealed for the first time the key factors influencing the subtypes of occupational stress among clinical female nurses, including age, years of working experience, and number of night shifts per month but also laid a solid theoretical foundation for an in-depth discussion of the intrinsic causes of occupational stress among clinical female nurses. In addition, these findings provide practical guidance and insights for tailoring and implementing individual intervention strategies for nursing practitioners on the basis of specific occupational stress subtypes.

Critically, our findings establish a clear gradient in PMS risk across these identified stress profiles. Nurses in the high-stress group reported significantly more severe PMS symptoms than those in the low-stress group. This pattern provides robust, person-centered evidence for the direct association between the overall experience of occupational stress and women’s gynecological health.

Among the three occupational stress subtypes identified, the moderate stress–slight overload care group accounted for the largest proportion, at 38.5%. This group of female clinical nurses had moderate overall stress but showed slight overload characteristics in specific dimensions, such as patient care and responsibility, workload and time management, and career development and identity. At the same time, there is also a certain amount of stress in the dimensions of management support and interpersonal relationships, the working environment, and resources. To address this, targeted interventions should focus on providing enhanced team support and practical stress-management training. This could include implementing peer-support programs or designated nursing mentors to alleviate care-related burdens, alongside workshops on time management, boundary setting, and cognitive-behavioral techniques to build personal resilience. Furthermore, optimizing the scheduling system through fair shift rotations and ensuring transparent pathways for professional development are crucial to address the specific overload dimensions identified. These measures are not only crucial for improving job satisfaction but may also play a preventive role in PMS management. By reducing chronic moderate stress, which can dysregulate hormonal pathways, such interventions could potentially lessen the severity of physical and psychological premenstrual symptoms in this substantial segment of the nursing workforce. On the other hand, the high stress-overload nursing and career development challenge group, which accounted for 23.2% of the total, faced significant psychological and physiological stress, mainly stemming from factors such as heavy tasks, complex patient care situations, and insufficient managerial support, which may also lead to psychological problems such as burnout, anxiety, and depression (31, 32). The finding that this group also had the highest PMS scores underscores a critical health disparity. The intense and multifaceted stress experienced by these nurses can be understood within the framework of chronic stress biology. Prolonged exposure to high demands likely leads to a dysregulated stress response, characterized by HPA axis hyperactivity and altered cortisol dynamics (1). This physiological state, in turn, may directly aggravate the neuroendocrine processes underlying PMS, including fluctuations in gonadal hormones and neurotransmitter activity, thereby increasing both the frequency and severity of premenstrual symptoms. Therefore, interventions for this group must prioritize structural and systemic reforms. These include: conducting regular, mandatory workload audits to redistribute excessive patient assignments; implementing protected time for complex care planning and mandatory breaks; and establishing clear, accessible pathways for career advancement and clinical ladder progression. The proposed stress management strategies are of dual importance: they are essential for preventing burnout and for breaking the cycle in which occupational stress worsens PMS, which, in turn, can impair coping abilities and amplify the perception of stress. In response to these issues, nursing managers should implement the aforementioned structural stress management strategies, optimize nursing loads through workload assessment tools, and support career development through mentorship programs to improve the quality of care and well-being of nurses (5).

In addition, the overall low-stress group accounted for 26.2% of the total sample. This group of female clinical nurses demonstrated lower stress levels at work and were able to cope better with daily work challenges and maintain good work–life balance. This group may benefit from effective team support, a reasonable workload, and good career development opportunities. However, it is worth noting that item 10 (no time to administer psychological care to patients) scored the highest of all the items. Despite the low overall group stress, this outlier suggests that we need to focus on the specific challenges faced by individual nurses. Possible reasons for this may include that the nurse is overloaded, is responsible for patients with particularly complex care needs, or lacks the necessary support and resources within the work environment to adequately carry out the psychological care of patients. The characteristically low PMS scores in this group substantiate the health benefits of a manageable work environment. To preserve this beneficial state, the primary goal should be to maintain the existing supportive environment while proactively monitoring for early signs of strain. Specifically, management should implement routine monitoring of high-load items through brief, periodic check-ins or surveys. This allows for the early identification and support of individuals who may be at risk of transitioning to a higher-stress profile, thereby protecting both their occupational well-being and gynecological health.

The study also revealed that female clinical nurses aged 18–30 years and with <10 years of work experience were more likely to belong to the moderate stress–slight overload care group and the high stress–slight overload nursing and career development challenge group. This demographic pattern highlights a high-risk subgroup that is doubly vulnerable: they are more prone to experiencing the most intense occupational stress and, as our results show, the associated heightened risk for severe PMS. This finding may reflect the stress and challenges faced by younger or less experienced nurses in coping with the demands of their jobs and personal career development. Consistent with the findings of this study, the study by Sun et al. indicated (33) that healthcare workers with 4–9 years of experience had a greater probability of being at risk of occupational stress than other age groups did, which further highlights the significant impact of the stage of experience accumulation on occupational stress. In addition, a study by Araújo et al. (34) focused on the occupational stress of nurses in mobile emergency services and revealed that a group of female nurses between the ages of 20 and 40 years suffers from high levels of occupational stress in the face of multiple challenges, such as precarious employment contracts, a lack of professional skills, limited control over their work, and a lack of motivation. In contrast, female clinical nurses with fewer than five night shifts per month were more likely to fall into the overall low-stress group. This is particularly relevant for PMS, as night shift work is known to disrupt circadian rhythms, which can directly exacerbate menstrual cycle irregularities and premenstrual symptoms. The lower PMS scores in the low-stress group may be partially mediated by this reduced night shift frequency (1). This difference may be related to the distribution of workload and maintenance of physiological rhythms; i.e., reducing the frequency of night shifts may have alleviated, to some extent, the physiological and psychological stress caused by night shift work. Studies have shown (1, 21, 35) that nurses who work night shifts for long periods have a significantly increased risk of cardiovascular disease, which may be caused by the continuous disturbance of physiological rhythms by night shift work. In addition, the excessive workload may lead to nurses becoming apathetic toward patients and their families (13).

The findings suggest that nurses in the high-stress-overload nursing and career development challenge group may be at greater risk for PMS, whereas nurses in the overall low-stress group demonstrated lower levels of associated symptoms, which is consistent with the findings of established studies showing that higher levels of stress are associated with a greater incidence of PMS. Specifically, a previous study by Wang et al. noted that increased stress correspondingly increases the risk of PMS (1). A study by Chen et al. showed (36) that exacerbation of PMS is closely related to occupational stress and anxiety and that the specific nature of the nursing work environment increases the susceptibility of the nursing population to these stressors. Nursing is usually accompanied by intense workloads, frequent night shift rotations, emergency responses, and complex patient care, which together constitute a high-stress environment. In this environment, nurses need to cope not only with physical fatigue but also with psychological and emotional stressors that may further exacerbate the symptoms of PMS. However, although occupational stress and anxiety are difficult to avoid completely in nursing, their negative effects can be mitigated to a certain extent by optimizing the working environment, providing psychological support, and implementing effective stress management strategies, thereby improving nurses’ physical and mental health (16). Therefore, this study not only deepens our understanding of the relationship between occupational stress and women’s health but also provides an important theoretical basis.

The strength of this study lies in its human-centered approach (30), which provides an in-depth exploration of the occupational stress levels among female clinical nurses. However, this study also has several limitations. First, the cross-sectional design precludes any inference of causality between occupational stress profiles, their predictors, and PMS. The associations observed here require validation through longitudinal or interventional studies to establish temporal precedence and causal direction. Second, the use of self-reported questionnaires for both independent and dependent variables may introduce common method bias, potentially inflating the observed relationships. Although procedural remedies including ensuring anonymity and using validated scales were employed, the potential influence of this bias cannot be fully ruled out. Third, the reliance on a convenience sample from a single hospital in Jiangsu Province severely limits the generalizability of the findings. The stress profiles, prevalence, and identified associations may not be representative of nurses in other regions, healthcare settings, or cultural contexts. Future research should employ multicenter, large-sample designs to enhance external validity. Additionally, the naming of the latent profiles, while data-driven, would benefit from further theoretical grounding and external validation in diverse samples.

## 5 Conclusion

The results of this study revealed significant group heterogeneity in occupational stress among female clinical nurses, which was manifested in three different profiles: the overall low-stress group, the moderate stress-slight overload care group, and the high stress-overload nursing and career development challenge group. These profiles not only differ in occupational stress levels but also significantly differ in demographic characteristics such as age, years of work experience, and the number of night shifts per month. These findings suggest that the distribution of occupational stress is closely related to nurses’ personal backgrounds and work environments, providing important clues for further understanding the mechanisms of occupational stress development. In addition, this study further revealed the potential impact of these occupational stress patterns on PMS, providing an important basis for understanding the relationship between different occupational stresses and PMS. Subsequent studies can explore how to develop individualized intervention strategies based on different occupational stress patterns to effectively alleviate the occupational stress of clinical female nurses, increase their job satisfaction and occupational stability, and provide a scientific basis for improving the quality of nursing care and promoting nurses’ occupational health.

## Data Availability

The raw data supporting the conclusions of this article will be made available by the authors, without undue reservation.
